# Differences in physical activity and mental function according to the employment status of elderly Japanese

**DOI:** 10.1002/1348-9585.12411

**Published:** 2023-06-22

**Authors:** Mami Ishizuka‐Inoue, Asuka Kawaguchi, Soshiro Kashima, Momoko Nagai‐Tanima, Tomoki Aoyama

**Affiliations:** ^1^ Human Health Sciences, Graduate School of Medicine Kyoto University Kyoto Japan

**Keywords:** elderly, employment, mental health, physical activity, self‐efficacy

## Abstract

**Objectives:**

In recent years, the employment statuses of the elderly have become more diverse, and it is important to investigate the differences in health status according to employment statuses. This study aimed to examine the differences in physical activity and mental function among elderly Japanese according to their employment status and to examine the differences between men and women.

**Methods:**

This cross‐sectional study used an online questionnaire. The participants were persons aged ≥60 years. Data on their sociodemographic indicators, employment status, physical activity, and mental function were collected. They were classified into six groups according to their employment status: being employed, completely retired, re‐hired at the same workplace, re‐hired at a different workplace, early retirement, and working at a job without a mandatory retirement age. Differences in the surveyed items according to employment status were compared using the Kruskal‐Wallis test.

**Results:**

The total number of participants in the analysis with complete responses was 1552 (1207 men and 345 women; mean age 67.8 ± 5.9 years). The results revealed that among men, those who were re‐hired at different workplaces had higher walking physical activity, and retirees and early retirees had longer sedentary time and lower sense of self‐usefulness. There was no clear difference among women according to their employment status.

**Conclusions:**

The results suggest that physical activity and mental function among older adults may differ according to their employment status, especially for men. Employment among the elderly may play an important role in maintaining their physical activity and mental function.

## 1 INTRODUCTION

In Japan, the legal mandatory retirement age is 60 years, and employment opportunities can be secured until the age of 65. In addition, in 2021, the law was amended to ensure that employment opportunities are available to people until the age of 70 (Act on Stabilization of Employment of Elderly Persons, Act No. 68 of May 25, 1971, Article 8, 9, and 10). The number of elderly workers is increasing every year, and according to a 2021 survey, 71.5% of those aged ≥60 and 50.3% of those aged ≥65 are employed.^1^ However, the percentage of irregular employment is reported to increase after the age of 60,^1^ and many workers are expected to change from regular to irregular employment when re‐hired beyond the mandatory retirement age.

In addition, some elderly people retire before the mandatory retirement age using the Early Retirement Incentive Program. Such programs provide preferential treatment to employees who retire before the mandatory retirement age by adding to their retirement benefits or treating them in the same way as those who retire at the mandatory retirement age. In a sample survey conducted by the Ministry of Health, Labor, and Welfare in 2021, 84 of the 166 companies with a mandatory retirement age reported having an early retirement incentive program.^2^

For the elderly, there are various working options: retiring, working, being re‐hired, or retiring early. Prior studies on the employment of the elderly have been limited to comparisons between workers and retirees and between before and after retirement conditions. In particular, studies on physical activity and mental health have been reported. One study conducted in Singapore reported that unemployed persons were less aware of their health status, partook in fewer physical activity, and were more likely to have dementia and lifelong depression than employed persons.^3^ One Spanish study found that employed people had higher self‐reported health level than retired people.^4^ One study conducted in the UK that focused on physical activity found that sedentary time decreased and walking time increased with retirement,^5^ in contrast with the results of the Singaporean study. One study conducted in Japan reported an increase in physical activity with retirement,^6^ supporting the findings of the UK study. Furthermore, the Japanese study mentioned above found that retirement led to improved self‐rated health and reduced psychological distress in men and women,^6^ in contrast to the aforementioned Singaporean and Spanish studies. A review of studies from several countries that examined the effects of retirement on health status found that retirement had a beneficial effect on mental health but its effects on physical health were inconsistent among the studies.^7^

Indeed, there is no unified view on the differences in physical activity and mental health between workers and retirees, and the results vary depending on the country the study is conducted in. Furthermore, while studies have simply categorized workers and retirees, none have categorized the elderly according to various workstyles in Japan today.

Moreover, perceived self‐efficacy has been reported to be positively correlated with mental health.^8^ Self‐efficacy is the degree of confidence in an individual's ability to carry out various actions and is considered important for carrying out tasks at work and in daily life.^9^ Additionally, self‐efficacy has been shown to be related to energy, depression, sleep quality, physical pain, and life satisfaction in the elderly,^10^ suggesting that improving self‐efficacy may be useful for mental as well as physical health. However, the relationship between workstyle and self‐efficacy in the elderly has not been examined. In addition, the feeling of being useful, similar to self‐efficacy, is an emotion defined in relation to others, i.e., being helpful to others or being appreciated by others.^11^ This sense of self‐usefulness has been used in research as one of the items in subjective well‐being scales.^12^ Recently, Ito et al. created a questionnaire focusing exclusively on self‐usefulness that showed good validity and reliability.^12^ The sense of self‐usefulness may be related to workstyle because it is a socially based emotion, in addition to simple self‐efficacy. However, the relationship between the sense of self‐usefulness and workstyle has not yet been investigated, and further studies are needed.

Moreover, when conducting research on workstyles in Japan, it is important to consider both men and women separately. In Japan, there is a gap in the employment rate between men and women,^13^ partly due to the historical belief that “men work and women do housework.” Thus, the purpose of this study was to examine the differences in physical activity and mental function of elderly Japanese according to their employment status as well as the differences between men and women.

## 2 METHODS

### 2.1 Study design and setting

**FIGURE 1:**
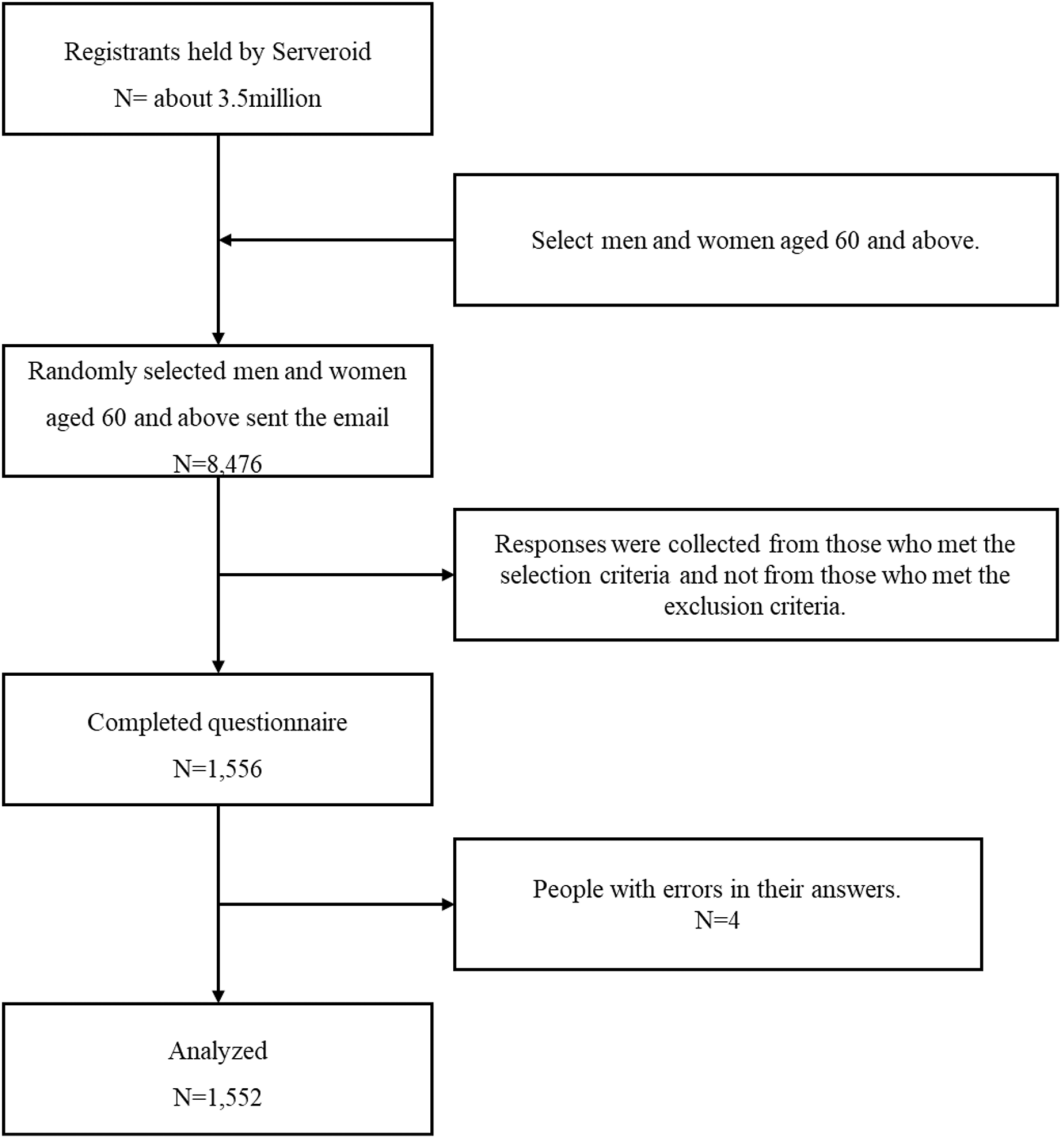
Recruitment flow.

A cross‐sectional online survey was conducted on July 29, 2022, involving individuals aged 60 years and above. The selection criteria were as follows: worked until age 60 or used the Early Retirement Incentive Program and retired before age 60. The exclusion criterion was having worked for an annual income of 1.3 million yen or less, which is the standard exemption by the Japanese social insurance system. The participants were recruited online by Surveroid (Marketing Applications Inc., Japan). Surveroid is an online research tool that has 3.5 million survey monitors, who are males and females in their 10s‐70s living in Japan.^14^ Approximately 75% of the monitors are residents of urban areas of Japan.^14^ The main analysis was a one‐way analysis of variance with six groups by employment status, with an effect size of *f* = 0.1, based on Cohen's standard effect size,^15^ an alpha error probability of .05, and a beta error probability of .2. The required sample size was calculated to be 1290 participants. Surveroid collects data at 20% above the required sample size to account for response errors or dropouts.^14^ Therefore, the target sample size was calculated to be 1548 participants. Of the 3.5 million registrants held by Serveroid, 8474 men and women aged 60 and above were randomly selected and sent survey requests via e‐mail. The purpose and methods of the study were explained at the beginning of the questionnaire. Submission of the questionnaire implied consent to participate in the study. The questionnaires did not ask for personal information such as names or email addresses. The participants received a reward upon completion based on their registration status in the Surveroid database. The eligibility of the completed questionnaires was checked, and the questionnaires from the participants who met the selection criteria were collected. In all, 1556 (18.4%) responses were received. Of these, four participants were excluded because of incomplete responses. The final number of participants was 1552 (18.3%) (Figure 1).

### 2.2 Measures

The online questionnaire included six domains, requiring 10 min for completion: (1) sociodemographic indicators, (2) employment status, (3) physical activity, (4) mental health, (5) self‐efficacy, and (6) sense of self‐usefulness.

#### 2.2.1 Sociodemographic indicators

The participants were asked about their sex, age, educational level, and marital status.

#### 2.2.2 Employment status

The participants were asked to select from six items regarding their current employment status: being presently employed (BE), that is, the person had not reached the mandatory retirement age of 65; completely retired (CR), re‐hired at the same workplace after retirement (RS), re‐hired at different workplaces after retirement (RD), early retirement (ER), and working at a job without a mandatory retirement age (WWR).

#### 2.2.3 Physical activity

Physical activity was assessed using the International Physical Activity Questionnaire Short Version (IPAQ‐SV).^16^ The IPAQ is a self‐administered questionnaire that measures physical activity, and the Japanese version was shown to be valid and reliable by Murase et al.^17^ Walking, moderate physical activity, vigorous physical activity, total physical activity, and sedentary time were calculated according to “Guidelines for the data processing and analysis of the International Physical Activity Questionnaire (in Japanese)”.^18^

#### 2.2.4 Mental health

Mental health was evaluated using the Japanese version of the World Health Organization Five‐point Well‐being Index (WHO‐5J).^19^ The validity and reliability of the WHO‐5J for use in the elderly were confirmed by Awata et al.^20^ The WHO‐5J consists of five questions about mood in daily life, with each item assessed on a 6‐point scale (0 = not at all, 1 = only occasionally, 2 = less than half the time, 3 = more than half the time, 4 = almost always, and 5 = always). The total score for the five items was calculated, with higher scores indicating a better mental status.

#### 2.2.5 Self‐efficacy

Self‐efficacy was assessed using the generalized self‐efficacy scale; the Japanese version was developed by Sherer et al.^21^, ^22^ The questionnaire consists of 23 items on social skills and professional competence, and each item is assessed on a 5‐point scale (1 = not at all applicable, 2 = not very applicable, 3 = neither applicable nor not applicable, 4 = fairly applicable, and 5 = very applicable). According to the developers' method, the total score of all items was calculated after reverse scoring the reverse items. The higher the total score, the higher the self‐efficacy.

#### 2.2.6 Sense of self‐usefulness

Sense of self‐usefulness was assessed using a scale with confirmed validity and reliability for older adults in Japan (Table 1).^12^ The questionnaire consisted of 12 items, and the respondents were asked to answer on a 5‐point scale (1 = not at all applicable, 2 = not very applicable, 3 = neither applicable nor not applicable, 4 = fairly applicable, and 5 = very applicable). The average score of all the items was calculated, with higher scores indicating a higher sense of self‐usefulness.

**TABLE 1:** Sense of self‐usefulness questionnaire.

Question items
I think I am appreciated by all around me.
I feel that my existence is recognized by all around me
I feel I am needed
I feel that people around me are interested in me
I am a source of emotional support to those around me
I am useful to society
I am an important member of society
I am needed by society
I have a role to play
I think people/pets around me would be in trouble without me
I think people/pets will miss me if I am not around

### 2.3 STATISTICAL ANALYSIS

Participant characteristics were analyzed using descriptive statistics. To examine differences in the measurements according to employment status, the participants were grouped according to employment status. After confirming the normality of each measurement item using the Shapiro‐Wilk test, we compared the differences between groups using one‐way ANOVA for normal distributions and the Kruskal‐Wallis test for non‐normal distributions. If the difference between the groups was significant, all pairs were compared using the Steel‐Dwass test. Additionally, we performed the same analysis separately for men and women to examine the differences in trends between the sexes. The significance level for the rejection of the null hypothesis was 5%. Statistical analyses were performed using JMP Pro version 16.0 statistical software (SAS Institute Japan Co., Japan).

## 3 RESULTS

### 3.1 Participants' characteristics

Of the 1552 participants, 1207 (77.8%) were men and 345 (22.2%) were women, with a mean age of 67.8 ± 5.9 years (range, 60‐92 years) (Table 2). The values for all measures are shown in Table 2. The results of the Shapiro‐Wilk test were all non‐normally distributed. Therefore, we used the Kruskal‐Wallis test for all tests of comparison of differences.

**TABLE 2:** Participant characteristics and index scores.

Variables	*N* (%)
Sex	
Men	1207 (78)
Women	345 (22)
Education level	
Middle school	35 (2)
High school	459 (30)
Technical school and junior college	202 (13)
University	795 (51)
Graduate school	61 (4)
Marital status	
Married	1275 (82)
Unmarried	277 (18)

Variables	
Age (years)	67.8 ± 5.9
PA (METs*min/week)	
Vigorous	80 (0–1600)
Moderate	0 (0–480)
Walking	396 (66–990)
Total	1233 (264–3155.25)
Sedentary time (min/day)	300 (120–480)
WHO‐5J	14.83 ± 5.84
Self‐efficacy	72.20 ± 11.12
Sense of self‐usefulness	3.11 ± 0.83

*Note*: Age, WHO‐5J, self‐efficacy, and sense of self‐usefulness are mean ± SD. PA and Sedentary time are median (interquartile range).

Abbreviations: METs, metabolic equivalents; PA, physical activity; WHO‐5J, World Health Organization Five‐point Well‐being Index.

The number of participants by employment status was 150 (10%) for BE, 527 (33%) for CR, 185 (12%) for RS, 154 (10%) for RD, 259 (17%) for ER, and 277 (18%) for WWR (Table 3). There was a significant difference in age according to employment status (Table 3). The BE group was significantly younger than all other groups, while the CR was significantly older than all other groups; the RS group was significantly younger than all groups except for the BE group; the WWR group was significantly younger than the RD group.

**TABLE 3:** Differences in physical activity and mental function by employment status.

	BE	CR	RS	RD	ER	WWR	*P*‐value
Total, *N* (%)	150 (10)	527 (33)	185 (12)	154 (10)	259 (17)	277 (18)	
Age	62.9 ± 0.4	71.1 ± 0.2	64.8 ± 0.4	68.1 ± 0.4	66.8 ± 0.3	66.6 ± 0.3	<.001**
PA (METs*min/week)							
Vigorous	0 (0–1250)	0 (0–1680)	240 (0–1440)	480 (0–1980)	0 (0–1680)	160 (0–1440)	.469
Moderate	0 (0–480)	0 (0–360)	0 (0–480)	0 (0–480)	0 (0–240)	0 (0–540)	.120
Walking	495 (66–1031.25)	396 (0–990)	396 (198–957)	792 (198–1386)	297 (49.5–792)	396 (66–767.25)	<.001**
Total	999 (280.5–3082.25)	1230 (198–3045)	1386 (396–2853)	1738.5 (550.5–4078.5)	876 (132–3213)	1386 (297–3333)	.038*
Sedentary time (min/day)	180 (60–360)	300 (180–480)	180 (95–400)	240 (115–480)	300 (180–500)	240 (120–435)	<.001**
WHO‐5J	15.00 ± 0.48	15.13 ± 0.25	14.29 ± 0.43	15.08 ± 0.47	14.32 ± 0.36	14.86 ± 0.35	.387
Self‐efficacy	72.57 ± 0.90	72.68 ± 0.48	70.61 ± 0.82	73.77 ± 0.89	70.77 ± 0.69	72.55 ± 0.67	.013*
Sense of self‐usefulness	3.30 ± 0.07	3.00 ± 0.04	3.13 ± 0.06	3.27 ± 0.06	3.01 ± 0.05	3.19 ± 0.05	<.001**

*Note*: Kruskal‐Wallis test. Age was significantly lower in BE than in all other groups, significantly higher in CR than in all other groups, significantly lower in RS than in all groups except BE, and significantly lower in WWR than in RD. Walking physical activity was significantly higher in RD than in CR, ER, and WWR. Total physical activity was significantly higher in RD than ER. Sedentary time was significantly longer in CR than in BE, RS, and WWR. ER was also significantly longer than all other groups except CR. Self‐efficacy was significantly lower in RS than in RD. Sense of self‐usefulness was significantly lower in CR than in BE, RD, and WWR. Age, WHO‐5J, self‐efficacy, and sense of self‐usefulness are mean ± SD. PA and Sedentary time are median (interquartile range).

Abbreviations: BE, being presently employed; CR, completely retired; ER, early retirement; METs, metabolic equivalents; PA, physical activity; RD, re‐hired at different workplaces after retirement; RS, re‐hired at the same workplace after retirement; WHO‐5J, World Health Organization Five‐point Well‐being Index; WWR, working at a job without a mandatory retirement age.

* <.05;

** <.01.

### 3.2 Differences in physical activity and mental function based on employment status

The results of comparison of physical activity and mental function according to employment status are shown in Table 3. We found significant differences in walking activity, total physical activity, and sedentary time according to employment status. Walking activity was significantly higher in the RD group than in the CR, ER, and WWR groups. Total physical activity was significantly higher in the RD group than in the ER group. Sedentary time was significantly longer in the CR group than in the BE, RS, and WWR groups, and in the ER group than in all other groups, except for the CR group. Regarding mental function, the WHO‐5J scores showed no significant differences between the groups. Self‐efficacy and sense of self‐usefulness differed significantly according to employment status. Self‐efficacy was significantly lower in the RS group than in the RD group. The sense of self‐usefulness was significantly lower in the CR group than in the BE, RD, and WWR groups.

### 3.3 Differences in trends, stratified by sex

The results of the subgroup analyses by sex are shown in Tables 4 and 5. Men showed significant difference by employment status in terms of age, walking activity, sedentary time, and sense of self‐usefulness. With respect to age, the BE group was significantly younger than all other groups; the CR group was significantly older than all other groups; the RS group was significantly younger than all other groups except for the BE group; and the WWR group was significantly younger than the RD group. The RD group had significantly higher levels of walking activity than the CR, ER, RS, and WWR groups. Sedentary time was significantly longer in the CR group than in all other groups except for the ER group, and in the ER group than in all other groups except CR group. The sense of self‐usefulness was significantly lower in the CR group than in the RD group, and in the ER group than in all other groups except for the CR group. These trends for men were similar to the overall trend. On the other hand, no significant differences were found among women across all measures, except age. The BE group was significantly younger than all other groups, except for the RS, and the CR group was significantly older than all other groups, except for the RD group.

**TABLE 4:** Differences in physical activity and mental function by men's employment status.

	BE	CR	RS	RD	ER	WWR	*P*‐value
Total, *N* (%)	104 (9)	438 (36)	167 (14)	142 (12)	165 (14)	191 (15)	
Age	63.0 ± 0.5	71.4 ± 0.2	64.8 ± 0.4	68.1 ± 0.4	66.9 ± 0.4	66.5 ± 0.4	<.001**
PA (METs*min/week)							
Vigorous	0 (0–1620)	0 (0–1920)	240 (0–1440)	480 (0–2220)	0 (0–1680)	240 (0–1680)	.512
Moderate	0 (0–480)	0 (0–360)	0 (0–480)	0 (0–480)	0 (0–240)	0 (0–720)	.070
Walking	396 (41.25–1138.5)	396 (0–1155)	396 (198–924)	767.25 (198–1386)	297 (49.5–990)	396 (66–792)	.001**
Total	999 (99–3276)	1336 (198–3218.25)	1374 (396–2853)	1831.5 (519.75–4196.5)	972 (126–3394.5)	1440 (297–3693)	.124
Sedentary time (min/day)	180 (67.5–360)	300 (180–500)	180 (120–400)	240 (115–485)	360 (190–600)	248 (120–480)	<.001**
WHO‐5J	14.61 ± 0.57	14.89 ± 0.28	14.35 ± 0.45	14.96 ± 0.49	13.42 ± 0.45	14.96 ± 0.42	.073
Self‐efficacy	73.25 ± 1.10	72.80 ± 0.53	70.84 ± 0.86	73.71 ± 0.94	70.89 ± 0.87	72.95 ± 0.81	.062
Sense of self‐usefulness	3.25 ± 0.08	2.92 ± 0.04	3.15 ± 0.06	3.25 ± 0.07	2.81 ± 0.06	3.11 ± 0.06	<.001**

*Note*: Kruskal‐Wallis test. Age was significantly lower in BE than in all other groups, and significantly higher in CR. Age was significantly lower in RS than in all groups except for BE, and significantly lower in WWR than in RD. Walking physical activity was significantly higher in RD than in CR, ER, RS, and WWR. Sedentary time was significantly longer in CR than in all other groups except for ER. ER was significantly longer than all other groups except for CR. Sense of self‐usefulness was significantly lower in CR than in RD. ER was significantly lower than all other groups except for CR. Age, WHO‐5J, self‐efficacy, and sense of self‐usefulness are mean ± SD. PA and Sedentary time are median (interquartile range).

Abbreviations: BE, being presently employed; CR, completely retired; ER, early retirement; METs, metabolic equivalents; PA, physical activity; RD, re‐hired at different workplaces after retirement; RS, re‐hired at the same workplace after retirement; WHO‐5J, World Health Organization Five‐point Well‐being Index; WWR, working at a job without a mandatory retirement age.

** <.01.

**TABLE 5:** Differences in physical activity and mental function by women's employment status.

	BE	CR	RS	RD	ER	WWR	*P*‐value
Total, *N* (%)	46 (13)	89 (26)	18 (5)	12 (4)	94 (27)	86 (25)	
Age	62.9 ± 0.8	69.6 ± 0.6	64.8 ± 1.3	67.8 ± 1.6	66.6 ± 0.6	66.8 ± 0.6	<.001**
PA (METs*min/week)							
Vigorous	40 (0–960)	0 (0–1440)	0 (0–1140)	0 (0–1080)	0 (0–1620)	0 (0–1200)	.998
Moderate	0 (0–480)	0 (0–380)	0 (0–420)	40 (0–1860)	0 (0–240)	0 (0–360)	.636
Walking	594 (198–1031.25)	396 (24.75–693)	371.25 (127.88–1089)	792 (618.75–1179.75)	297 (33–717.75)	396 (66–693)	.054
Total	1021.5 (519.75–2293)	1032 (198–2377.5)	1440.5 (336.38–2841.75)	908.5 (716.25–3591)	816 (198–2805)	1207.5 (288.75–2564.25)	.736
Sedentary time (min/day)	180 (60–360)	240 (120–300)	165 (60–375)	210 (105–300)	300 (180–420)	240 (120–360)	.156
WHO‐5J	15.74 ± 0.84	16.35 ± 0.60	13.72 ± 1.34	16.42 ± 1.64	15.90 ± 0.59	14.64 ± 0.61	.218
Self‐efficacy	71.04 ± 1.60	72.08 ± 1.15	68.4 ± 2.56	74.33 ± 3.14	70.56 ± 1.12	71.67 ± 1.17	.647
Sense of self‐usefulness	3.42 ± 0.11	3.39 ± 0.08	3.00 ± 0.17	3.48 ± 0.21	3.37 ± 0.08	3.35 ± 0.08	.375

*Note*: Kruskal‐Wallis test. Age of BE was significantly lower than that of all groups except for RS, and significantly higher of CR than that of all groups except for RD. Age, WHO‐5J, self‐efficacy, and sense of self‐usefulness are mean ± SD. PA and Sedentary time are median (interquartile range).

Abbreviations: BE, being presently employed; CR, completely retired; ER, early retirement; METs, metabolic equivalents; PA, physical activity; RD, re‐hired at different workplaces after retirement; RS, re‐hired at the same workplace after retirement; WHO‐5J, World Health Organization Five‐point Well‐being Index; WWR, working at a job without a mandatory retirement age.

** <.01.

## 4 DISCUSSION

The purpose of this study was to examine the differences in physical activity and mental function of elderly Japanese according to their employment status as well as the differences between men and women. Our results revealed differences in physical activity, sedentary time, self‐efficacy, and sense of self‐usefulness depending on the employment status. Subgroup analyses by sex revealed that physical activity, sedentary time, and sense of self‐usefulness differed by employment status for men, but not for women. The Labour Force Survey conducted in 2021 reported that 25% of those aged 65 and older were employed in some way.^23^ No results were found that summarized the employment status of employees aged 60 and older. Although this survey covered employees aged 60 and over, which is the legal retirement age, many companies actually ensure employment opportunities up to the age of 65. Therefore, the percentage of those working is considered to be slightly higher (50%) than the Labor Force Survey results. The proportions of men and women were 78% and 22%, respectively. According to the White Paper on Gender Equality 2021, 55% of all employed persons are men and 45% are women.^13^ However, we believe that the low percentage of women responding is unavoidable because only 40% of the non‐working population comprises women aged 60 years and above^23^ and because of the historical background context of the small number of working women in Japan. In addition, we found significant differences in age based on the working style. Since the retirement age has legally been extended to 65, and the government has mandated that employers take measures to ensure a continuous employment system and employment opportunities up to age 65, workers who had not reached the retirement age and those who were re‐hired at the same workplace tended to be younger. Furthermore, since we did not set an upper age limit for the participants, it is likely that some retirees were older than those who were currently working.

The results of this study revealed that the trends for men were similar to the overall trends, indicating that physical activity and mental functioning differed by employment status. Conversely, there were no differences in employment status among women. This result may have been influenced by attitudes toward women's workstyles in Japan where women often take on child‐rearing and housework roles. In the 1992 survey, only 26.3% of the respondents said they would prefer to continue working in their profession throughout their lives, even after having children.^13^ In the 2019 survey, the percentage of women at work increased to 63.7%. However, traditional ideas may be prevalent among those aged >over 60 years of age, the target population for this study. It is possible that child‐rearing and family roles are more important to women than their work roles. No differences in employment status were observed among women.

Among men, walking activity was found to be higher among those who were re‐hired at a different workplace compared to those who retired and early retired and re‐hired at the same workplace and worked at a job without a retirement age.

A review of the motivational factors for physical activity levels among adults aged ≥60 years summarizes the motivational factors from three perspectives: intrapersonal, interpersonal, and environmental.^24^ In the intrapersonal domain, improvements in physical condition and psychological states such as positive perception of physical activity and positive self‐image are reported as motivational factors, while in the interpersonal domain, being social is reported as the most important motivational factor.^24^

Those who were re‐hired at different workplaces were found to have significantly higher sense of self‐usefulness than those who were completely retired. In addition, although not significantly different, those who were re‐hired at the same workplace and those who were working at a job without retirement age had lower self‐usefulness scores than those who were re‐hired at different workplaces. In other words, it is possible that those who were re‐hired at different workplaces were motivated to engage in physical activity because of their positive self‐image. It is also possible that those who are re‐hired at different workplaces experience an increase in social involvement, such as relationships at their new workplace, and the increased social involvement may motivate them to engage in physical activity. Thus, those who work in different workplaces have more factors that may motivate them to be physically active than those who choose other workstyles. It is also possible that the individuals who were re‐hired at different workplaces may have been engaged in jobs that involved physical activity. This study was unable to investigate the type of occupation that led to increased physical activity; hence, this aspect should be examined in the future.

It was also found that retirees had significantly longer sedentary time than those who were still working. Previous studies have reported an increase and a decrease in the effect of retirement on physical activity, and no unified view has been reached.^3^, ^5^, ^6^ A review of the association between retirement and physical activity also concluded that exercise and leisure physical activity increase with the transition to retirement.^25^ However, no clear pattern emerged with respect to overall physical activity.^25^ The short version of the IPAQ used in our study asked about weekday sedentary time.^16^ While retirees are not expected to differ significantly in how they spend their weekdays and holidays, it is likely that many people work during the weekday. Therefore, it is thought that workers have less sedentary time on weekdays due to work, which may have caused a significant difference in the sedentary time between the workers and retirees.

A previous study reported a median sedentary time of 4.7 h per day for adults in 62 countries and of 4.9 hours per day when limited to high‐income countries.^26^ The median sedentary time observed in the current survey was 5 h for retirees and 6 h for early retirees, which is longer than the time shown in the previous survey. The median sedentary time for those still working ranged from 3 to 4.1 h. High levels of sedentary time have been reported to be unfavorably associated with cognitive function, depression, functional disability, physical activity levels, and physical health‐related quality of life.^27^ Therefore, an approach to encourage retirees to reduce their sedentary time may be necessary.

Self‐usefulness was lower among retirees than among those working at different workplaces, and lower among early retirees than among those working. The sense of self‐usefulness is an emotion that is generated in relation to other people, for example, by being useful to others or appreciated by others.^11^ Low sense of self‐usefulness may be related to the fact that retirees tend to think that they have completed one of their social roles by retiring.

Additionally, as a result of the overall analysis only, those who were re‐hired at different workplaces had significantly higher self‐efficacy than those who were re‐hired at the same workplace. A previous study conducted in Japan reported that working in the same workplace after mandatory retirement may lead to a decline in cognitive function, while those who work at a different workplace after retirement or who have retired may retain their cognitive function by being exposed to a new environment and stimuli.^28^ Additionally, they noted the possibility that not all retirees are exposed to new environments and stimuli.^28^ Generalized self‐efficacy, as measured in this study, is suggested to influence the expectation of being able to adaptively handle not only specific situations but also new, situations not yet experienced.^22^ Thus, it is likely that those rehired in different workplaces after mandatory retirement are more exposed to new environments and stimuli and thereby experience a greater sense of coping with these situations, which may also contribute to their higher self‐efficacy. On the other hand, the cross‐sectional study cannot explain the causal relationship, and it is possible that high self‐efficacy may have allowed the participants to choose a different workplace after retirement.

This study had four main limitations. First, it was conducted in Japan and the results cannot be generalized to other countries since working styles and attitudes toward work differ from country to country. Additionally, this was a cross‐sectional study and could not explain the causal relationships. It is necessary to conduct a longitudinal study, following the participants before they reach the mandatory retirement age. Furthermore, this study involved online monitors registered with Surveroid. As such, the selection may have been biased toward participants with relatively high levels of education who were familiar with the online environment and resided in urban areas. Therefore, further studies are needed with a larger number of participants with various demographic backgrounds to strengthen the results of this study. Finally, there is a lack of consideration regarding income. Previous studies have reported that higher income, but not employment status, is associated with a higher quality of life.^29^ In the future, it will be necessary to consider labor income, unearned income, and household income as well.

## 5 CONCLUSION

This study examined the differences in physical activity and mental function according to employment status among elderly Japanese individuals. The results revealed that among men, those who were re‐hired at different workplaces had higher walking activity than the others, retirees and early retirees had longer sedentary time, and early retirees had lower sense of self‐usefulness. There was no difference in physical activity and mental functions among women depending on their employment status. As the working styles of the elderly become more diverse, their physical activity and mental functioning may differ depending on their workstyle, especially for men. Employment among the elderly may play an important role in maintaining their physical activity and mental function.

## AUTHOR CONTRIBUTIONS

Mami Ishizuka‐Inoue, Asuka Kawaguchi, Soshiro Kashima, Momoko Nagai‐Tanima, and Tomoki Aoyama conceptualized the ideas and collected the data. Mami Ishizuka‐Inoue analyzed the data and drafted the original draft. Asuka Kawaguchi, Soshiro Kashima, Momoko Nagai‐Tanima, and Tomoki Aoyama drafted, edited, and reviewed the final version. All authors approved of the final version.

## CONFLICT OF INTEREST STATEMENT

The authors declare that there is no conflict of interest.

## DISCLOSURE

*Approval of the research protocol*: This study was approved by the Medical Ethics Committee of Kyoto University (R3600). *Informed consent*: The purpose and methods of the study were explained at the beginning of the questionnaire. Submission of the questionnaire implied consent to participate in the study. *Registry and registration no. of the study/trial*: N/A. *Animal studies*: N/A.

## Data Availability

The data that support the findings of this study are available from the corresponding author upon reasonable request.
